# Eruption Cyst in the Neonate

**DOI:** 10.5005/jp-journals-10005-1485

**Published:** 2017-02-01

**Authors:** Alline J de Oliveira, Maria LG Silveira, Danilo A Duarte, Michele B Diniz

**Affiliations:** 1 Professor, Department of Pediatric Dentistry, Tocantins Institute President Antonio Carlos, Tocantins, Brazil; 2 Professor, Department of Pediatric Dentistry, Tocantins Institute President Antonio Carlos, Tocantins, Brazil; 3 Professor, APCD School of Dentistry, Sao Paulo, Brazil; 4 Professor, Post Graduate Program in Dentistry, Cruzeiro do Sul University Sao Paulo, Brazil

**Keywords:** Congenital, Eruption cyst, Neonate.

## Abstract

The pediatric dental approach to the oral cavity of newborns requires special attention, as many aspects are unique and peculiar to this period of life. It is important that pediatricians and pediatric dentists be aware of the characteristics within normal newborn patterns and prepared to make a correct diagnosis of abnormalities at early stages. Congenital eruption cysts (ECs) are rarely observed in newborns, as at this stage of a child’s life, tooth eruption is unusual. This study reports a case of EC treated successfully by monitoring of the lesion, without any surgical procedure. In the 4th month, the lesion had completely regressed, and the deciduous central incisors had erupted without problems. The clinical and radiographic monitoring of ECs in newborns seems to be a satisfactory management procedure, similar to what is recommended for older children.

**How to cite this article:** de Oliveira AJ, Silveira MLG, Duarte DA, Diniz MB. Eruption Cyst in the Neonate. Int J Clin Pediatr Dent 2018;11(1):58-60.

## INTRODUCTION

Eruption cysts are located superficially on the crown of a tooth at the eruption stage, slightly before emerging into the oral cavity.^1,2^ The cyst appears as a bluish, translucent, elevated, compressible lesion in the shape of a dome in the alveolar ridge, as one of the local changes during teeth eruption.^3^ Discomfort is rare; however, the presence of this lesion can hinder or even harm the appearance of teeth in the oral cavity.^4^

This change involves predominantly deciduous man-dibular central incisors and permanent first molars.^5^ On radiographic examination, it is difficult to distinguish the space between the EC and the tooth, as both are closely linked with the soft tissues of the alveolar crest.^6,7^

This report aims to describe a rare case of congenital EC treated successfully by monitoring of the lesion, without any surgical procedure or tooth extraction.

## CASE REPORT

**Fig. 1:**
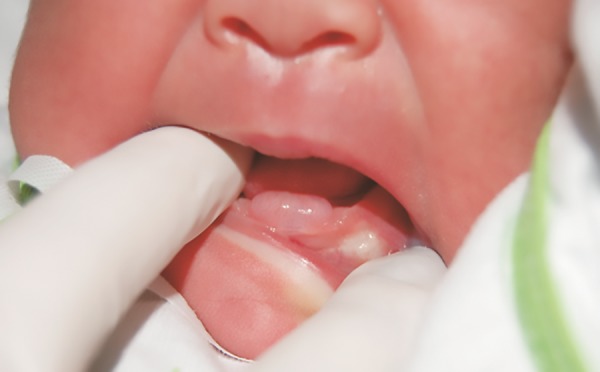
Gingival bulge in the anterior mandible, equivalent to the presence of central incisors

At the Dom Orione Maternity Hospital in the city of Araguaina, Tocantins, Brazil, a male infant newly born through natural child birth, full-term, weighing 3600 gm, presented with gingival bulging in the anterior mandible, equivalent to the presence of central incisors (Fig. 1).

**Fig. 2:**
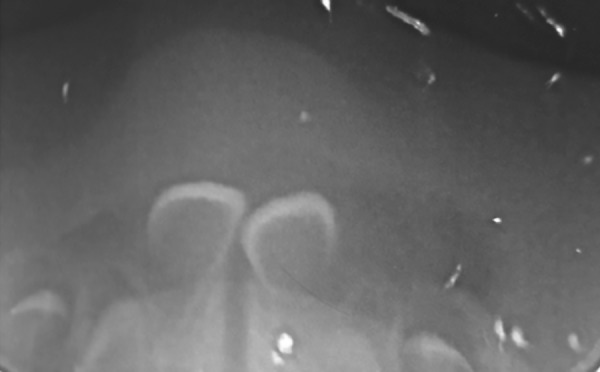
Radiographic appearance of the reported area

Physical examination revealed an exophytic lesion 2 cm in diameter, compressible, flaccid, and yellow, leading to an initial diagnosis of “congenital EC.” The newborn exhibited no feeding problems or other complications. A radiograph of the mandibular anterior occlusal revealed the superficial placement of the deciduous central incisors (Fig. 2). The procedure of choice, based on the patient’s age and the clinical diagnosis, was monitoring of the injury. Regardless of this initial decision, the possibility of surgical intervention was not discarded if there was no spontaneous regression.

**Fig. 3:**
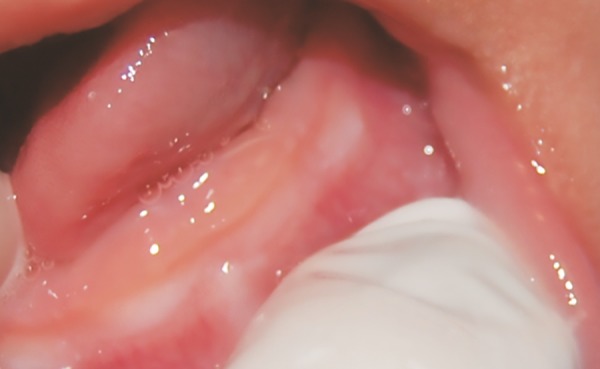
One-month follow-up, showing a significant reduction in lesion size

**Fig. 4:**
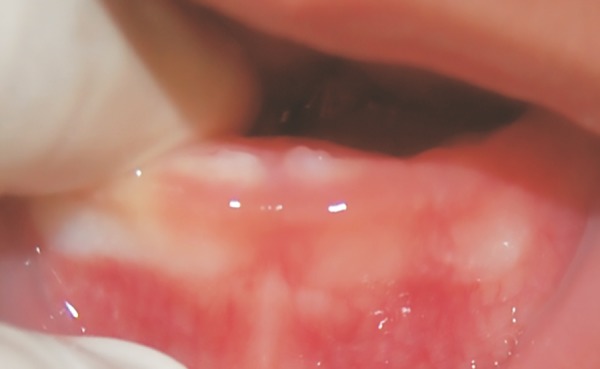
Four months follow-up, showing normal eruption of the deciduous central incisors

Follow-up visits were scheduled after 15 days and monthly thereafter. After approximately 1 month, the lesion size had decreased, and the color changed from yellowish to normal gingiva color (Fig. 3). By the 4th month, the lesion had completely disappeared, and the deciduous central incisors erupted without problems (Fig. 4). Follow-up radiographs showed normal root development of the central incisors. The patient is still under control, exhibiting a normal eruption sequence and complete regression of the initial lesion.

## DISCUSSION

Eruption cyst is a pathological condition found in the oral cavity during childhood, represented by swelling of the soft tissue located on the alveolar ridge, adjacent to the erupting tooth.^5,6,8^ The origin of the EC is still debated. Certain authors attribute its origin to degenerative changes of the reduced enamel epithelium after the end of amelo-genesis or suggest that the cyst develops from the remnants of the dental lamina that coats the erupting teeth.^5^ Regardless of the precise etiology, the term EC is correctly used when the affected tooth is located in the soft tissues lining the alveolar bone during tooth eruption. When the tooth is surrounded by bone, the same lesion should be referred to as a dentigerous cyst, according to the classification adopted by the World Health Organization.^3,4,9^ In this case study, the radiographs showed normal deciduous central incisors with no evidence of bone pathology.

Clinically, the EC appears as a circumscribed, floating, often translucent volumetric increase in the alveolar impeller at the site of tooth eruption. When the cir-cumcoronal cystic cavity contains blood, the swelling appears purplish or dark blue and is called an eruption hematoma.^7^

The clinical findings observed in this case are characterized by a fluctuating and compressible swelling suggestive of fluid retention, resembling a mucocele. However, secondary salivary glands are not found in the alveolar impeller, excluding this hypothetical diagnosis.^4^

Although most reports of ECs are in the first decade of life, only a few studies have shown this type of lesion in newborns.^10^

Aleman Navas et al^3^ reported a case of neonate presenting a lesion 2 × 2 cm in diameter, exophytic, soft, yellowish, and compressible in the anterior mandibular region. The diagnosis of EC was made by clinical, radio-graphic, and histopathological examination. At 4 months, the lesion had completely disappeared, and the deciduous central incisors had erupted without problems.

Ricci et al^4^ described a case of EC in a newborn, in which clinical follow-up was adopted. Forty-five days after the first consultation, the lesion had nearly disappeared. Complete regression was observed after 60 days, and 4 months after the initial examination, the central incisors had already erupted, showing small hypoplastic areas on the incisal edges.

Peters and Schock^11^ reported a case of a newborn presenting two symmetrical lesions in the anterior dome of the mandible alveolar ridge. Treatment was established, and periodic follow-up visits were scheduled. At 2 months, both incisors were completely erupted.

Although the EC cannot be detected on radiographic examination due to the lack of bone involvement, radiography is recommended to evaluate the morphology of the affected tooth and its surrounding bone.^3,5,6^ Radiographic examination in this case revealed normal deciduous central incisors, with no evidence of bone pathology.

Eruption cysts may be associated with natal or neonatal teeth and have been classified as mature natal or neonatal teeth when the tooth is almost or fully developed and has relatively good prognosis to be maintained or as immature natal or neonatal teeth when the tooth has incomplete or nonstandard structure, resulting in poor prognosis.^3^ When poorly implemented, the natal or neonatal tooth is dangerous to the newborns, as it can loosen during breastfeeding and be aspirated; thus, extraction is indicated.^3,12,13^ In the clinical case in question, the central incisors were palpable in the cyst; however, the decision to preserve them was made because they did not present hypermobility, did not interfere with feeding, and did not cause discomfort in the mother or the child.^3^

The treatment options for EC have been thus far no procedure and monitoring,^3,4,9,11^ marsupialization,^10^ or surgical extraction of the involved tooth.^3,5,10^ When these cysts prevent the eruption of a deciduous tooth, marsupi-alization may be considered. Extraction is indicated when the teeth present mobility or interfere with lactation.^11^ In this case of congenital EC, the treatment of choice was monitoring of the lesion, without any surgical intervention or tooth extraction.

In newborns, clinical and radiographic monitoring of the EC seems to be adequate, similar to the procedure recommended for older children.
